# Blood-Based Biomarkers for Managing Workload in Athletes: Perspectives for Research on Emerging Biomarkers

**DOI:** 10.1007/s40279-023-01866-5

**Published:** 2023-06-21

**Authors:** Nils Haller, Thomas Reichel, Philipp Zimmer, Michael Behringer, Patrick Wahl, Thomas Stöggl, Karsten Krüger, Perikles Simon

**Affiliations:** 1 Department of Sports Medicine, Rehabilitation and Disease Prevention, Johannes Gutenberg University of Mainz, Mainz, Germany; 2 Department of Sport and Exercise Science, University of Salzburg, Salzburg, Austria; 3 Department of Exercise Physiology and Sports Therapy, Institute of Sports Science, Justus-Liebig-University Gießen, Giessen, Germany; 4 Division of Performance and Health (Sports Medicine), Institute for Sport and Sport Science, TU Dortmund University, Dortmund, Germany; 5 Department of Sports Sciences, Goethe University Frankfurt, Frankfurt am Main, Germany; 6 Department of Exercise Physiology, German Sport University Cologne, Cologne, Germany; 7 Red Bull Athlete Performance Center, Salzburg, Austria

## Abstract

At present, various blood-based biomarkers have found their applications in the field of sports medicine. This current opinion addresses biomarkers that warrant consideration in future research for monitoring the athlete training load. In this regard, we identified a variety of emerging load-sensitive biomarkers, e.g., cytokines (such as IL-6), chaperones (such as heat shock proteins) or enzymes (such as myeloperoxidase) that could improve future athlete load monitoring as they have shown meaningful increases in acute and chronic exercise settings. In some cases, they have even been linked to training status or performance characteristics. However, many of these markers have not been extensively studied and the cost and effort of measuring these parameters are still high, making them inconvenient for practitioners so far. We therefore outline strategies to improve knowledge of acute and chronic biomarker responses, including ideas for standardized study settings. In addition, we emphasize the need for methodological advances such as the development of minimally invasive point-of-care devices as well as statistical aspects related to the evaluation of these monitoring tools to make biomarkers suitable for regular load monitoring.

## Key Points

**Table:** 

A variety of novel blood-based biomarkers such as cytokines, chaperones, enzymes and other inflammatory signaling molecules are sensitive to acute and chronic exercise load and potentially useful for the monitoring of athlete training load.
Omics, RNA and DNA approaches as well as genetic testing could further improve athlete workload management.
Longitudinal study designs will shed light on acute and chronic training load responses to assess the suitability of emerging biomarkers for regular load monitoring. Methodological advances such as the development of point-of-care devices, as well as overcoming statistical challenges, are critical for the further progress of load monitoring with biomarkers.

## Introduction

Biomarkers (i.e., “indicators of normal biological processes, pathogenic processes, or responses to an exposure” [1]) have seen an upsurge in so-called personalized medicine, that is, procedures individually tailored to patients. They have great potential to be an objective complement to other screening methods in the diagnosis and prognosis of diseases, therapeutic decisions, as well as in the assessment of therapeutic success. For instance, concentrations of biomarkers have been shown to predict mortality or diagnose disease severity in certain conditions [2, 3]. Recent developments are moving from single biomarker measurement to multiple marker approaches to determine a whole range of biological measures. Artificial intelligence (AI) could be utilized to help analyze this data and assist physicians to make informed decisions about treatment options [4].

Transferring this perspective to the field of elite sports, ‘treatment options’ could be interpreted as the prescription of personalized training load and recovery. Blood-based biomarkers, which in principle are able to objectively reflect training load, fatigue, and recovery needs, are already being applied by practitioners to facilitate decision making and to ensure an individualized load management (i.e., prescription, monitoring, and adjustment of workload [5]) aimed at optimizing performance and avoiding injury [6, 7]. However, established biomarkers such as creatine kinase (CK) or lactate, while sensitive to training load and both convenient and quick to measure with point-of-care (POC) devices, primarily capture a specific physiological domain when measured solely in the absence of other markers. This means that practitioners in professional sports settings need to capture multiple markers to holistically assess the training response and consequently manage athlete workloads. High chronic training loads, body composition, as well as delayed peaks in concentrations may further lead to misinterpretations [8–10]. In the search for other reliable alternatives, a variety of novel biomarkers (e.g., CD163, heat shock proteins [HSP], cell-free DNA [cfDNA], blood cell ratios) have shown marked increases after standardized exercise settings [11–14], providing potential added value for load management.

To date, the cost and effort of measuring innovative parameters, such as cfDNA, HSP or cytokines, on a regular basis are still high, making them inconvenient for monitoring purposes. Due to evolving technology, these biomarkers may soon become relevant to sports medicine practitioners through POC devices. Many studies in this area also aim to determine a generalizable resilience or trainability profile of an athlete that may be relevant throughout their career, e.g., genetic markers for ligament injury [15]. Underlying all of these approaches in sports is a similar rationale to the clinical setting, which is the assessment of biomarkers to individualize treatment.

This current opinion article discusses innovative and load-sensitive biomarkers followed by advances in biotechnology, such as omics approaches, that enable the assessment of a variety of biomarkers and biosignatures. The section on practical applications focuses on methodological and statistical considerations, and ideas for future study designs to establish emerging biomarkers for load management in sports science.

## Evidence and Usefulness of Emerging Biomarkers for Workload Management

Many researchers have increased their efforts to identify new robust surrogate biomarkers to help in the management of training load [16, 17]. The goal is to identify biomarkers that represent appropriate and reliable responses to training load, reflect recovery cycles and regeneration processes, and thus are expected to make an important contribution to the field of load management in professional sports settings.

A frequently used starting point in recent years has been the immune system. Physical activity induces a systemic immune response manifested by leukocytosis, a shift in the proportion of leukocyte subpopulations, and the release of numerous pro- and anti-inflammatory cytokines [18]. Some of these molecules have a close relationship to metabolic changes during exercise or belong to the class of myokines, which act at the immunological level in addition to metabolic signaling pathways [19]. These immunological markers are very sensitive to acute exercise, depending, among other factors, on the duration and intensity of the load, with partial regulation also observed depending on the type of exercise [20, 21]. Some of these markers are classified as chemokines, such as chemokine-ligands, while others are classified as enzymes, such as myeloperoxidase [22]. The potential use of these proteins as biomarkers in exercise settings would also be of interest, as immunological markers indicate differential disturbances in physiological homoeostasis or tissue integrity. For example, some markers are regulated depending on the level of muscle damage, while others have dependencies on, for example, neuroimmunological processes, energetic deficit, or heat production [21, 22].

We understand that blood reflects only a small fraction of immunological processes, but these are quite well studied. Accordingly, immunological markers can provide an estimation of the internal load at different physiological levels and indicate regeneration processes. Markers of oxidative stress show a close connection to the immune response. Physical activity induces an increased level of reactive oxygen species (ROS). Accordingly, more products of oxidative stress are released, which can also be detected directly or indirectly in the blood [23]. Here, some markers are currently in focus that reflect the level of oxidative stress in the context of athletic exertion and the subsequent recovery cycles. Markers of the stress proteome, such as HSP, have a relationship to both immune changes and oxidative stress. For example, blood levels of HSP70/72 and HSP90 respond very sensitively to physiological stressors, such as endurance exercise, and are also quickly regulated back after the end of the exercise. Interestingly, HSP70 increases were shown to depend on intensity and duration with evidence of changes in resting concentrations after intensified training periods [14]. In line with these findings, recent studies have demonstrated the importance of Ca^2+^-binding proteins of the S100 group as possibly useful biomarkers in sports. S100 proteins represent a class of calcium-binding proteins that are sensitively released depending on the exercise load [24], showing increases after long-lasting endurance exercise with S100 Calcium Binding Protein B increasing mainly after running but not cycling exercise [25, 26]. In addition, Irisin is released in response to exercise as a result of proteolytic cleavage of FNDC5 protein present in the membrane of myocytes. The exact physiological mechanism of release is not yet completely understood [27], however, chronic training may lead to decreased levels of circulating irisin [28]. Another important category represents the field of adenosine triphosphate (ATP) metabolism catabolites, such as ammonia, hypoxanthine, or xanthine. In this respect, the combined measurement of lactate, ammonium, and hypoxanthine was shown to indirectly reflect changes in energy status during exercise [29]. In addition, hypoxanthine is discussed as being a promising marker for training status and a predictor of sport performance in athletes [30].

Of note, there are many other metabolites being discussed as suitable biomarkers in sports. The significant strain on the metabolism during exercise induces significant blood concentration changes after exercise in the composition of the plasma metabolome. For example, lipids and lipid-like substances are mobilized into the blood during long-lasting endurance exercise [31]. Metabolites can also provide information about the training status, as the metabolome of trained athletes changes according to the training adaptation at the metabolic level. Accordingly, some metabolites associated with cardiopulmonary fitness have already been identified, such as several acyl-alkyl-phosphatidylcholine species [32], while others are more prevalent in strength athletes, such as phosphatidylcholines [33]. Table 1 outlines relevant exercise response biomarkers that could potentially help practitioners monitor and manage athlete loads in the future.

**Table 1:** Emerging biomarkers with potential release mechanisms, interpretation of concentrations changes and their association with training load

Category	Biomarker	Release into circulation during exercise	Interpretation of increased concentrations after exercise	Association with exercise load
Cytokine	Interleukin 1 receptor antagonist (IL-1Ra)	Monocytes, macrophages, neutrophils, hepatocytes, synovial fibroblasts, mast cells [34]	Anti-inflammatory; indicator for inflammatory tissue damage [35]	Increase with endurance (eccentric and concentric) exercise [36, 37], 40 min of running at 95% of HR at IAS, followed by 20 min at 110% HR at IAS, and long-lasting endurance exercise [12, 19, 35, 38]
Cytokine	Interleukin 1 beta (IL-1β)	Macrophages and epithelial cells [39]	Exercise-induced inflammatory response [40]	Increase with long-lasting endurance exercise [19, 35, 37]
Cytokine	Interleukin 6 (IL-6)	T-helper cells 2, monocytes, and macrophages [41]	Pro-inflammatory; stimulates the immune response to trauma or other tissue damage [12, 42, 43]	Increase with long-lasting cycling endurance [44] and acute running exercise [19, 37, 42]
Cytokine	Interleukin 8 (IL-8)	Monocytes, endothelial cells, and fibroblasts [43, 45]	Pro-inflammatory; has an inflammatory effect and plays a role in angiogenesis in skeletal muscle; induces chemotaxis in activated immune cells, and phagocytosis [43]	Increase with endurance exercise (ultra-endurance exercise, 3 h of running/cycling) [43]; 40 min of running at 95% of HR at IAS, followed by 20 min at 110% HR at IAS [12]
Cytokine	Interleukin 10 (IL-10)	T-cells, B-cells, macrophages/monocytes, dendritic cells, and neutrophils [41, 46]	Anti-inflammatory; inhibits proinflammatory processes [46, 47]	Increase with long-lasting endurance exercise [19, 37]; 40 min of running at 95% of HR at IAS, followed by 20 min at 110% HR at IAS [12] as well as high-intensity intermittent exercise [48]
Cytokine	Interleukin 15 (IL-15)	Monocytes, macrophages, keratinocytes, epidermal skin cells, fibroblasts, various epithelial cells, bone marrow stromal cells, nerve cells, and dendritic cells [49]	Pro-inflammatory; due to mechanical load and muscle damage, IL-15 regulate T and natural killer cell activation and proliferation and is also responsible for muscle development and glucose metabolism in skeletal muscles [43, 45]	Increase with endurance exercise (30 min of running at 70% VO_2max_) [50]; 40 min of running at 95% of HR at IAS, followed by 20 min at 110% HR at IAS [12]
Cytokine	Interferon-gamma (IFN-γ)	Natural killer cells and T-helper cells 1 [41]	Exercise-induced inflammatory response [37]	Increase with long-lasting endurance exercise [37, 51]
Cytokine	Irisin	Skeletal muscle [52]	Mediator of exercise-induced energy metabolism [52]	Increase with acute speed/strength and endurance exercise protocols [52, 53], chronic training load associated with lower circulating irisin levels [28]
Cytokine	CC-chemokin-ligand-2 (CCL-2)	Monocytes, T cells, and dendritic cells [43]	Indicator for skeletal muscle damage and inflammation [43]	Increased in skeletal muscle after eccentric exercise, but not after concentric exercise [43, 54]
Enzyme	Myeloperoxidase (MPO)	Neutrophils and monocytes [55]	Exercise-induced inflammatory stress [55, 56]	Increase with short endurance exercise and after 3 days of intense cycling at rest [40, 57]
Chaperone	Heat shock protein 70 (HSP70)	Various cell types such as necrotic cells, lymphocytes, natural killer cells [14]	Exercise-induced inflammatory stress [14, 58]	Increase with acute and chronic exercises, primarily in endurance sports [14, 59, 60]
Chaperone	Heat shock protein 90 (HSP90)	Various cell types such as necrotic cells, lymphocytes, natural killer cells [14, 61]	Exercise-induced inflammatory stress [14, 62]	Increase with acute and chronic exercises, primarily in endurance sports [14, 63]
Other inflammatory signaling molecule	S100 calcium binding protein A8/A9 (S100A8/A9)	Myeloid cells and neutrophils [57, 64]	Exercise-induced inflammatory response [57, 64]	Increase with long-lasting endurance exercise [64–67]
Other inflammatory signaling molecule	S100 calcium binding protein B (S100B)	Astrocytes [26]	Exercise-induced impact in the brain; exercise-induce muscle damage [25]	Increases after endurance exercise, primarily running but not cycling exercise [25, 26]
Other inflammatory signaling molecule	Cluster of differentiation 163 (CD163)	Monocytes and macrophages [68]	Anti-inflammatory signaling activity; protect mechanisms initiated under conditions of oxidative-radical burden [68]	Increase after a habitual loading microcycle [20] or after long-lasting endurance exercise [68]
Other inflammatory signaling molecule	Thiobarbituric acid reactive substances (TBARS)	Product of lipid peroxidation [69]	Exercise-induced oxidative stress response [12, 42]	Increase after acute high-intensity interval training, and after severe resistance training over several weeks at rest; decrease after anaerobic exercise [70–72]
Other inflammatory signaling molecule	Hypoxanthine	Product of ATP degradation [30], release probably from skeletal muscles and/or myocardium [73]	Exercise-induced energetic stress [30]	Increase after sprinting [74], high-intensity or moderate-intensity exercise with intensity and duration as key variables [30, 75, 76]. Changes in concentrations at rest and changes in the acute exercise response during different training phases [30]
Other inflammatory signaling molecule	Cell-free, circulating deoxyribonucleic acid (cfDNA)	Granulocytes [77]	Exercise-induced aseptic inflammation [78]	Substantial increases during intermittent and endurance exercise [11, 79]; some evidence of increases due to chronic training load [78, 80]

*HR* heart rate, *IAS* individual anaerobic threshold, *VO*_*2max*_ maximal oxygen consumption

These markers were selected as they exhibit clear regulation from homeostasis by acute exercise and/or regular training, as well as a re-regulation to baseline during recovery. Initial conclusions about their usefulness are thus already possible, as temporal changes in the course of acute load-recovery cycles and also short-term cumulative training cycles have been shown (e.g., [11, 12, 20]). In the long term, the extent to which such markers reflect adaptive processes, for example by being associated with changes in cardiopulmonary fitness, is of particular interest [81]. Furthermore, for some markers, it is not understood to what extent these markers are differentially regulated during different types of exercise, such as strength or endurance training. Knowledge of sex-specific characteristics or classifications relevant to training status is not yet available and points to further limitations that need to be explored in the validation phases.

Most studies in this area were conducted in the context of acute exercise (Table 1), suggesting the use of biomarkers immediately after cessation of exercise to assess the acute physiological response in combination with parameters of external workload. In addition to evaluating training load, biomarker responses can provide additional insight, such as an estimation of the athlete's risk of disease as indicated by various cytokine responses after exercise (e.g., in IL1-Ra, IL-10) [82]. However, there is a need for easy-to-use instruments, such as POC devices, that allow practitioners to measure these biomarkers in a timely and simple manner. In addition, marker-specific obstacles need to be considered or overcome (e.g., low specificity, venous blood needed) before they can be regularly used for load monitoring. Section 4 will therefore address methodological aspects of biomarker development.

It should be noted that there are other physiological domains that we have thoroughly investigated. For example, proteomics approaches have identified fitness- or body fat-associated biomarkers such as leptin [83]. However, our focus has been on load-sensitive biomarkers of immunology rather than fitness or anthropometry, as we consider the latter to be less relevant for load management. Another interesting area of research could be cardiac biomarkers such as NT-proBNP or cardiac troponin, which have shown load-dependent patterns during exercise [84, 85]. As new biomarkers are identified, bioanalytical methods for measuring biomarkers continue to evolve, as discussed in Sect. 3.

## Bioanalytical Approaches with a Focus on Protein, Free-Circulating Nucleic Acids and Genetic Biosignatures

### Omics—Deciphering Bio-Signatures with a Multiple Marker Approach

Exercise induces a multilevel and complex physiological stimulus that is reflected at the level of gene expression, epigenetic processes, protein synthesis, energy metabolism, and the associated metabolome [86]. Sophisticated large-scale analytical methods to quantify gene expression (transcriptomics), proteins (proteomics), lipids (lipidomics) and metabolites (metabolomics) in different organs and tissues, in the context of exercise mainly in the muscle and blood, are now available to identify biomarkers that define different status of stress and recovery cycles or physiological adaptation processes. The so-called multi-omics profiling can even couple such technologies to analyze the interrelationships of the individual levels. Such technologies have also been used in sports and have significantly developed the choreographic interaction of different physiological levels [87].

Omics technologies need to be coupled with appropriate bioinformatic methods so that the truly relevant ones can be identified from the multitude of possible targets. Pathway and network analyses are an essential part of the bioinformatics analysis, which can strengthen the basic scientific understanding of the regulation of the markers. Such approaches have evolved in recent years to pathway and network-based biomarker analysis, which has a particular focus on discovering panels of markers that can serve as a biosignature rather than singular biomarkers. Bioinformatic databases have supported the functional analysis and interpretation of results [88]. Accordingly, today’s omics technologies can be valuable contributors to the identification of biomarkers, or biosignatures, and their physiological classification in the context of sports.

However, this approach should be understood as the first step in the complex process of biomarker research. Multi-omics profiling can, as a first step in identifying biomarkers, highlight those candidates within a network that have the greatest potential to be more robust to individual differences or to environmental factors other than training load. This idea can be illustrated by a study from Nieman et al. [57], who attempted to identify candidate biomarkers through a proteomics approach for functional overreaching over 3 days of extreme stress. Of nearly 600 proteins, over 70 proteins were identified that increased during the following recovery period at rest. Finally, the authors suggested the application of the identified biomarker panel in a more sophisticated training study with additional monitoring tools to examine the potential of these markers for predicting overtraining in athletes.

### Specific Approaches for Protein and Free Nucleic Acid Detection

Proteins are considered key biomarkers that allow monitoring of the training process. Due to their multiple functional roles as enzymes, cellular signaling molecules, co-factors, and neurotransmitters, many of these factors reflect load and recovery as well as adaptation processes. High-throughput analyses, such as those possible using multiplex assays or mass spectrometry, can provide indications for the identification of potential candidate proteins as a first step [16, 89].

In analogy to proteins, DNA or RNA are complex macromolecules that can be studied inside or out of cells as cfDNA [90] or circulating RNA (cirRNA). As for protein analysis, mass spectrometry gains importance in DNA and RNA analysis since it is not only capable of identifying post-translational protein modifications [91] but also nucleotide modifications of functional and physiological relevance. For proteins in fluids, enzyme-linked immunosorbent assay (ELISA) are used in laboratories, while derived from this principle, lateral flow immunoassays are used at POC. Lateral flow assays can nowadays also be used to analyze DNA or RNA at POC and techniques were developed during the COVID-19 pandemic to do this semi-quantitatively using the cell phone [92]. For high-throughput medical diagnostics of several different proteins or nucleic acids to be analyzed simultaneously in one sample, the principles of ELISA have been combined with flow cytometry.

Together with proteins, nucleic acids belong to a group of circulating macromolecules that can be subjected to covalent modifications in addition to their amino acid or base sequence and can therefore contain essential qualitative information in addition to their quantity. In analogy to typical glycosylation, phosphorylation, or citrullination of amino acids, DNA-bases can be methylated carrying epigenetic information [93]. While amino acid modifications are mostly indicators of a different conformational state, functional status, or stability, methylation of DNA can for certain sequence parts be cell-type specific and may therefore reveal the origin of DNA [93]. At present, there is a rapidly evolving field of so-called liquid biopsy by analyzing circulating nucleic acids in blood [94].

At the DNA level, quantification of cfDNA is a putative forward approach for monitoring acute [11, 79] and chronic [78, 80] exercise load. Quantitative analysis in combination with qualitative information has just been able to confirm the origin of cfDNA from cells of the hematopoietic lineage [95]. Cell-type specific epigenetic analysis of cfDNA has revealed that most of the DNA released during exercise is released within minutes from neutrophils [77]. Thus, unlike versatile cirRNA, cfDNA is the lead molecule for a specific process that is initially triggered with the onset of movement, that is, neutrophil activation. Increases of cfDNA, therefore, fall into the category of markers attributed to neutrophil activation that have been described as a prominent pathway affected during acute exercise in several omics approaches and a recent multi-omics approach [87]. Analysis of DNA by a highly sensitive detection technology has furthermore confirmed that the majority of DNA released by neutrophils is indeed free-floating in the bloodstream and not associated with or incorporated in extracellular vesicles [96, 97], while the full complexity of mRNA, long non-coding RNA, or microRNA analysis is not yet well investigated or even understood. Of note, in the field of cirRNA analysis, small differences in the analytical procedures can lead to unpredictably different outcomes. CirRNA can be protected from rapid decay by extracellular vesicles, which in turn are subject to significant change upon the onset of exercise [97, 98].

### Genetic Testing—The Promise of Once in a Lifetime Individualization

In contrast to previous approaches, genetic testing already plays a prominent role in load management, whereby a one-time measurement can already be relevant for the entire career. The determination of, for example, injury propensity [15, 99, 100] could be used to identify at-risk individuals and prescribe individualized exercise programs to counteract this injury propensity.

In recent years, the Genome-Wide Association Study approach has enabled a detailed understanding of the importance of specific genetic polymorphisms for sport-specific performance [101]. Basic research on gene polymorphisms and their association with phenotypic traits relevant in sports has generated evidence that specific genetic polymorphisms can affect training responses, the ability to regenerate, and the susceptibility to injury [99]. For example, IGF-1R 275124 A>C rs1464430 polymorphism was shown to be represented in endurance athletes and PPARGC1A polymorphism was also shown to be related to endurance performance. Furthermore, the RR genotype of ACTN3 R577X polymorphism, the C allele of IGF-1R polymorphism and the gene variant FTO T>A rs9939609 and/or their AA genotype showed a relation to muscle strength while gene variants of the MMP group (rs591058 and rs679620) and the COL5A1 rs13946 polymorphisms are associated with increased susceptibility to injury in athletes [99]. However, most of these studies are associative studies, which limits their practical value for designing training programs.

Thus, it seems particularly reasonable to proceed to interventional studies using genetic information. One of the first studies that used genetic testing to differentiate a training program was conducted by Jones et al. [102]. In this study, an algorithm of 15 different single nucleotide polymorphisms (SNPs) was developed to determine a power/endurance score ratio. The SNPs used included variants of the genes *ACE*, *ACTN3*, *ADRB2*, *AGT*, *BDKRB2*, *COL5A1*, *CRP*, *GABPB1*, *IL6*, *PPARA*, *PPARGC1A*, *TRHR*, *VDR*, and *VEGFA*. One group of athletes performed an 8-week strength training program aligned by genetic profile to be either more intense or of greater volume, while the control group performed exactly the training that did not match their genotype. Although the study can be critiqued methodologically in some aspects, the initial evidence generated showed that pre-intervention genetic profiling and assignment to a training program leads to a favorable outcome in terms of explosive power and aerobic fitness.

In relation to the release of circulating markers, genetic testing has a potentially growing importance. For some circulating markers, such as CK, there are high responders and low responders, which is most likely due to genetic polymorphisms. Accordingly, testing of such SNPs prior to the actual measurement of the markers may help to classify athletes with respect to their responder assignment in the future. For this purpose, causal links between the presence of individual or multiple SNPs still need to be established in future studies [103].

However, the extent to which genetic testing is finding its way into sports could also be a cause for concern. There has been a recent surge in commercial direct-to-consumer genetic testing without the involvement of a physician. Athletes and coaches are naturally focused on implementing, for example, effective training strategies to optimize performance, which may make this cohort particularly susceptible to such testing, believing that those results will contribute to improved performance outcomes [104]. Pickering and Kiely [105] found in 110 athletes and 133 practitioners that about 10% of both groups had already made use of such tests. However, when genetic testing is not conducted by experienced personnel, this may lead to misinterpretation and potentially serious data security issues and raise questions about whether athletes undergoing such procedures are actually fully informed in terms of purpose, possible results, and ramifications [104, 106]. In comparison with other tests, genetic testing may accidentally deliver an outcome that is as a stand-alone not valid enough for diagnostics but provides an outcome that should prompt further medical diagnostics. If the tests are not initiated by the athletes themselves, but by a sports authority, and are thus not exclusively voluntary, they could undoubtedly be classified as unethical [104, 107]. The fact that most genetic studies have been conducted in people of European ancestry [108] also appears to be problematic as the predictive performance may not be relevant for people of other ancestries.

In summary, genetic testing in sports has yielded interesting findings but, unlike disease-related genetic testing, it is still in its infancy. To date, neither tests for talent identification [107, 109] nor tests for exercise prescription or injury prevention [106] seem to have sufficient predictive value.

## Considerations for Researchers in Upcoming Studies

In this section, we highlight topics to further develop load management: (i) biomarker development, (ii) methodological advances, and (iii) statistical considerations.

### Biomarker Development

Biomarker development typically proceeds in three phases, that is, discovery, verification, and validation [110]. Biomarker discovery phase is primarily concerned with identifying a set of potential marker candidates in a given experimental design, using approaches such as omics as described above [40, 111, 112] to select potentially suitable load-sensitive markers for future studies. Verification is regularly performed by repeated measurements. In proteomics, for example, candidate biomarkers are subjected to additional analysis to verify their identity and expression in the same samples. The remaining identified markers can then be tested for sensitivity, specificity, and repeatability in a manner similar to the discovery phase (analytical validation). Finally, in the clinical validation (i.e., ‘qualification’ [110]), the identified reliable and robust markers [12] can be applied to similar interventions [110]. At each stage, the markers of interest are further narrowed, leading to an intensified testing of biomarker performance for clinical use [110].

The potential for biomarker discovery also arises from the primary use of markers in clinical settings and their transfer to sports. Historically, cfDNA has been introduced as a marker for cancer screening [113] but also showed a pronounced acute response after strenuous exercise [11] and first evidence of indicating overtraining status [78]. In addition, well-known ratios such as the neutrophil-to-lymphocyte or testosterone-to-cortisol ratios were also introduced as promising tools for load monitoring. In this respect, associations with overtraining or anabolic adaptation to training have already been observed [13, 114, 115].

Regarding further evaluation of biomarkers, standardized test–retest settings seem reasonable for assessing the reliability of a biomarker and its response to an acute load [12]. An adequate post-exercise observation period provides information on the reregulation to baseline and the biological half-life time of these biomarkers. Amateur athletes or youth squads of elite teams [7] can be used as a study population, with a subsequent transfer to the domain of professional athletes. Accurate characterization of participants taking into account high and low responders, the documentation of potential confounding variables due to the effects of environmental factors, and the determination of the external load can provide insights into dose–response patterns [14, 116, 117].

**Fig. 1:**
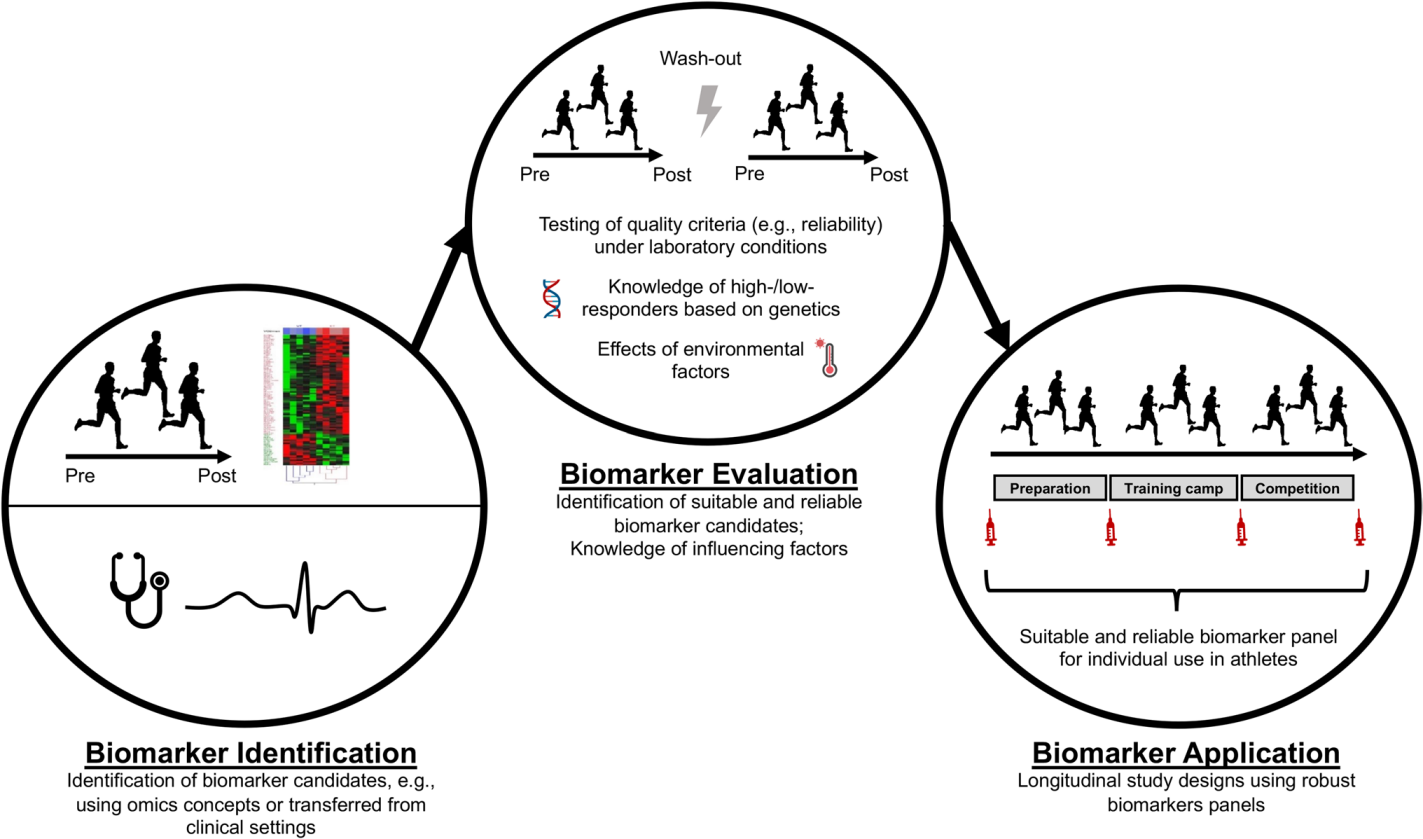
Phases of biomarker development. Ideally, the identification phase (through omics approaches or transferred from clinical settings) is followed by an assessment using standardized exercise settings. Finally, the biomarker is then applied in longitudinal study designs

Finally, longitudinal study designs are efficient in observing the potential of biomarkers to ‘forecast’ an event such as an injury or illness, as well as to reveal the acute and chronic response to an unusual intervention, such as training camps. Repeated blood sampling at rest and post-exercise—in combination with established monitoring tools—can help to assess the training load and the corresponding biomarker responses while establishing individualized reference values [118] with a conclusion on the principal suitability of the biomarker (panel) (Fig. 1). Furthermore, insights can be gained about gender specifics, and effects of training volume and training status [119].

### Methodological Considerations

Since repeated blood sampling is necessary to generate reliable data, the development of convenient and efficient measurement methods is important to avoid excessive burden on athletes and to keep the amount of blood collected low [120]. Various cytokines in capillary blood from the finger are regulated similarly to those in venous blood. Regarding blood plasma from the earlobe, there is less data on biomarker regulation [121, 122] although the measurement is possible in principle [123], which would greatly improve the accessibility of the biomarker and would not require specialized personnel. Such a continuous transition to capillary or even noninvasive saliva or urine collection has also been demonstrated for other markers such as cfDNA [124–126]. Concurrently, the development and establishment of measurement methods is challenging, as concentrations in different fluids are usually difficult to compare.

Another methodological aspect is that results of biomarker analyses are often available only with a delay due to the sophisticated technology involved. This provides only a retrospective view of the training session, which limits the possibilities of load management for practitioners [127]. To fulfill the requirement for rapidly available results, POC devices, mobile tools, or microarray-based screenings are necessary but may have limitations in terms of limit of detection and sensitivity. Therefore, it is of great importance to introduce innovative technologies with higher sensitivity and in miniaturized form to the market, such as the already presented protein microarrays for evaluation on a picogram level [128] for validated, reliable, load-, injury-, or overtraining-associated marker panels. Currently, various manufacturers are developing POC devices that can measure biomarkers, such as HSP, or various cytokines, such as IL-6, IL-8 or IL-10, highly efficiently in small amounts of plasma [129–131]. They work in the form of miniaturized protein microarray-based assays or as molecular processors and have made technological progress, particularly because of the COVID-19 pandemic [132].

### Data Processing and Statistical Concerns

To obtain a comprehensive picture of the athlete, a holistic marker panel that covers various aspects of performance, muscle status, and even nutritional aspects is recommended [16], with the downside of large data sets occurring for monitoring purposes. Biomarker, performance, and questionnaire data lead to myriad ways to analyze these data. In the case of biomarkers, the literature has shown that the simple cause-effect model is not sufficient to understand the biology of an athlete [133]. The response after exercise is complex and vast, with interconnected processes and pathways.

It is generally recognized that AI, and especially machine learning (ML), can supplement basic statistical approaches when it comes to (i) data processing and visualization but also for (ii) planning and decision making [134]. ML algorithms can make predictive capability both more accessible and more accurate using existing registries and big databases together with new variables. These algorithms increase the prediction power by calibrating the equations from the different distributions of the analyzed variables, which hold the potential to change decision making dramatically and optimize individual outcomes [135, 136]. These approaches are making great progress in personalized medicine, where computer-aided analysis of biomarker data and/or imaging techniques are used to improve, for instance, a cancer patient's therapy [137]. Biomarker data have proven helpful, although decision making is still delayed due to the increasing complexity of novel biomarkers [138]. Thus, high throughput computational approaches are fundamental to create accurate prediction tools with clinical applicability and translation that hold highly impactful potential.

ML is expected to be increasingly used in professional sport settings in the future [134], with the aim that ‘artificial trainers’ will support coaches in their decision making in workload management. This is already common in the automated analysis of tools such as wearable heart rate monitors, which record daily activity, visualize data, and provide personalized training recommendations [134]. In the case of athlete monitoring, first steps to predict injuries have been taken in both individual [139] and team sports [140]. ML approaches were shown to be principally capable of predicting the internal load (RPE) in Australian Football players with an artificial-neural-network analysis (ANN) with session distance as a predictor [141]. Furthermore, ANN and least absolute shrinkage and selection operator revealed decelerations as an important variable to predict the RPE in soccer players [142]. A meta-analysis on ML approaches for injury prediction showed that research has already identified several predictor variables ranging from sleep quality and genetic variables to external load variables, such as the distance covered [143]. However, the quality of the included studies was moderate to low.

Accurate prediction of athlete responses to a planned training session and knowledge of injury risk factors allows practitioners to effectively prescribe individualized training loads [141]. There has been a focus on external load variables, which have been integrated into the ML models as predictor variables. The integration of biomarker data could further improve models, such as for injury prediction [144]. Finally, the use of multi-marker approaches and their evaluation with ML can also help to identify the suitability of biomarkers [145] for load management, as large data sets and aggregation of biomarkers with all other data can reveal previously unrecognized patterns.

Finally, the use of additional monitoring tools not only results in additional data, but also requires financial and human resources that must be carefully weighed against the associated benefits of a serially measured biomarker to ultimately decide on its use [127]. If a sports organization is able to perform, analyze, and interpret the biomarker in a nonobstructive and frequent manner, this may represent an additional benefit. If these additional resources are not available, organizations and practitioners are well advised to use easy-to-use tools such as questionnaires for the determination of the psychophysiological exercise response.

## Conclusion and Outlook

Promising methodological approaches could soon transfer robust and valid biomarkers from the medical field to practical application in sports science. Many biomarkers have been shown to be capable of reflecting different aspects of exercise load (Table 1). Ideally, these biomarkers will be accessible in a short time using minimally invasive blood collection methods and a miniaturized POC device, although this is still in the future for some of the mentioned biomarkers. In addition, several markers have yet to prove their full potential compared with established markers such as CK or urea, for which POC devices are already available.

The collected biomarker data should be analyzed in combination with athlete characteristics, training data, and further monitoring tools using appropriate statistical approaches, to create a personalized athlete profile or at least an athlete cluster leading to individualized load management approaches. Ideally, these approaches may help to estimate the individual training responses, adverse events such as injuries, and resilience factors in athletes. Algorithms that incorporate training load recommendations can serve as a solid foundation for practitioner decision making. The ultimate goal is to combine ML with practitioner expertise to take load management to a new level [142].
